# Glenohumeral internal rotation deficit in non-pitcher overhead athletic athletes: case series analysis of ten athletes

**DOI:** 10.1016/j.amsu.2020.08.050

**Published:** 2020-09-08

**Authors:** Andri MT. Lubis, Rizky P. Wisnubaroto, Ermita I. Ilyas, Nadia NPPS. Ifran

**Affiliations:** a Department of Orthopaedic & Traumatology, Faculty of Medicine Universitas Indonesia, Jakarta, 10430, Indonesia; b Department of Orthopaedic & Traumatology, Cipto Mangunkusumo General Hospital, Jakarta, 10430, Indonesia; c Department of Physiology, Faculty of Medicine, Universitas Indonesia, Central Jakarta, Jakarta, 10430, Indonesia

**Keywords:** Glenohumeral internal rotation deficit, Throwing athletic athlete, Shoulder

## Abstract

**Background:**

The glenohumeral internal rotation deficit (GIRD) is diagnosed when there is a loss of 20° of internal rotation compared to the contralateral shoulder. This condition has already been well described in a group of throwing athletes, i.e. baseball pitchers. However, athletic athletes such as javelin throwers, discus throwers, hammer throwers, shot putters may also be susceptible to this condition. Reports are lacking to recognize these symptoms as GIRD for these group of athletes. We aim to evaluate these subgroups of athletes for the possibility of GIRD.

**Materials and methods:**

We examined ten athletes (javelin, hammer throwers, and shot putters) for signs of GIRD. Signs of loss of internal rotation were assessed by measuring shoulder range of motion (internal rotation and external rotation) in supine position and posterior shoulder tightness test. Complaints of shoulder pain, evidence of scapular malposition, inferior medial border prominence, coracoid pain and malposition, and dyskinesis of scapular movement (SICK) scapula, posterior shoulder flexibility test were examined.

**Results:**

The athletes had a mean training period of 3.8 years. One athlete had complaints of mild pain on their dominant shoulder. Two athletes had GIRD (20° and 25°) with no posterior shoulder tightness. Three athletes had posterior shoulder tightness, but normal total shoulder ROM (195°, 180°, and 185°). Three athletes had increased external rotation (105°, 100°, 125°). No subjects had scapular dyskinesia nor SICK scapula syndrome. All athletes had normal total shoulder ROM.

**Conclusion:**

Glenohumeral internal rotation deficit could be present in non-pitcher overhead athletics athletes.

## Highlights

• Repetitive throwing motion on the shoulders of athletic athletes may cause glenohumeral internal deficit.
• The condition is commonly reported in baseball pitchers, but less so in other overhead athletes.
• We found shoulder related complaints are found in athletic athletes but no deficit in the total shoulder range of motion.

## 1 Introduction

The glenohumeral internal rotation deficit (GIRD) is a condition that is generally described as a loss of internal rotation in throwers. This disorder is defined as loss of 20° of internal rotation compared to the contralateral shoulder and occur during the late cocking and early acceleration phase. This process has been thought as an adaptive process of the shoulder to compensate for the repeated stresses exerted during a throwing motion [[1], [2], [3]]. This condition mostly appeared and described in details in athletes with overhead activities i.e. baseball pitchers. (see )

**Table 1:** Athletes profile.

No	Age (years)	Sex	Duration of training (years)	Sport	Dominant arm	Symptoms
1	19	M	5	Hammer throw	Right	–
2	18	M	5	Hammer throw	Right	–
3	15	M	2	Shot put	Right	–
4	17	M	2	Shot put	Right	–
5	14	F	2	Hammer throw	Right	–
6	18	F	4	Hammer throw	Right	–
7	21	M	7	Discus throw	Right	–
8	18	M	4	Discus throw	Right	–
9	17	F	2	Javelin	Right	Right ant. Shoulder pain
10	23	M	5	Javelin	Right	–

**Table 2:** Physical examination findings.

No	Total ROM						Posterior shoulder flexibility (Tyler test)			SICK Scapula rating
	Right shoulder			Left shoulder			Dominant Arm			
	IR	ER	Total ROM	IR	ER	Total ROM	Difference in Cm	Right or left	Degrees (1 cm–5′)	
1	70	120	190	90	100	190	1	Right	5	1
2	90	105	195	90	90	180	4	Right	20	0
3	60	90	150	85	95	180	3	Right	15	1
4	100	100	200	100	90	190	2	Right	10	0
5	75	120	195	90	105	195	1.5	Left	7.5	0
6	70	130	200	60	120	180	2	Right	10	0
7	90	90	180	90	85	175	4	Right	20	0
8	90	100	190	95	105	200	1	Right	5	0
9	95	90	185	100	90	190	5	Right	25	2
10	90	100	190	90	100	190	2.5	Right	12.5	1

Keys: IR (internal rotation), ER (external rotation), ROM (Range of motion); unless specified all numerical values pertains to degrees in rotation.

However, overhead activities are not only exclusive to baseball pitchers. Other sports also lend their physical feats by using their shoulders in an extreme fashion. For example, javelin throwers have been reported to have deficiency in internal rotation and tennis players were reported with deficit and additional internal impingement [4,5].

The purpose of this case series was to determine if similar pathologies could be found clinically in high performing throwers in athletics, (javelin throwers, discus throwers, and shot putters). Similar symptoms have been found clinically but reports are lacking to formally recognize these symptoms as GIRD for these specific group of athletes.

## 2 Materials and methods

We examined ten professional national athletic throwers, javelin throwers (2 athletes), short putters (2 athletes), hammer throwers (4 athletes), and discus throwers (2 athletes) from the National Athletics Federation at a teaching hospital. We took the patient's history; complaints of shoulder stiffness, the need for prolonged warm-up, and posterior shoulder pain being the focus of our examination [6].

**Fig. 1:**
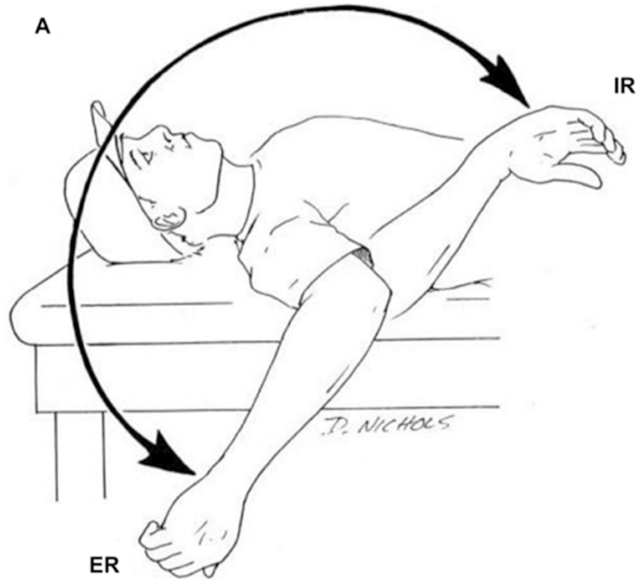
Examination of shoulder internal and external rotation with the athlete put in a supine position. Note: ER: external rotation, IR: internal, total ROM: summation of IR and ER. Taken from: Reinold MM, Gill TJ. Current concepts in the evaluation and treatment of the shoulder in overhead-throwing athletes, part 1: Physical characteristics and clinical examination. Sports Health. 2010; 2 (1):39–50 [21].

**Fig. 2:**
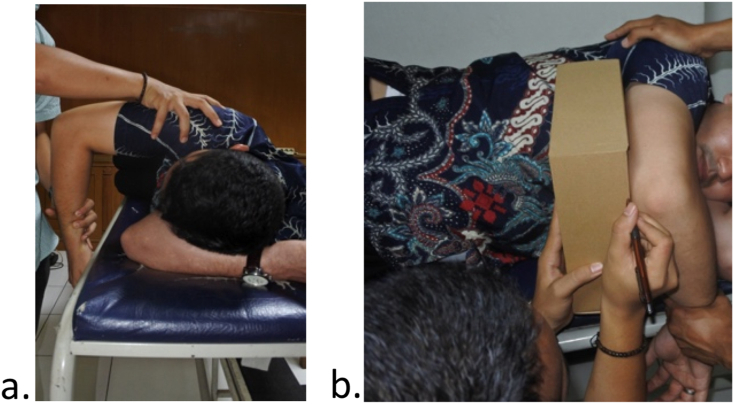
The Tyler test to measure posterior shoulder tightness. (A) The patient is positioned correctly on his side, the examiners arm is stabilizing the scapula, the shoulder is adducted maximally. (B) A recorder marks the square distance of the medial epicondyle falls on a plinth [7].

Two authors, with one being a senior orthopaedic sports surgeon and an orthopaedic surgeon, examined the same patient independently to reduce interobserver variation. We then followed the method that was described by Rose and Noonan and Burkhart et al. to diagnose the patient [1,2]. The athlete was put in a supine position and we assessed the total rotation motion (TRM) of the shoulder. We defined it as a summation of passive external rotation (ER) and internal rotation (IR) (Fig. 1.). Posterior tightness is then assessed using the method described by Tyler et al. [7] The patient was put in a lateral decubitus position with the scapula stabilized, and perpendicular to a plinth (Fig. 2.). The medial epicondyle is then measured in centimeters to the plinth. Every 1 cm loss of adduction correlates with a 5° loss of internal rotation. Therefore, a 4 cm deficit compared to the non-throwing arm would be equivalent to a 20° of loss of IR and a diagnosis of GIRD [1,7].

**Fig. 3:**
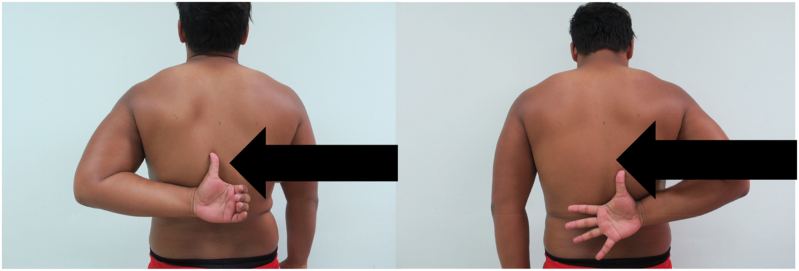
Adduction and internal rotation examination. The tip of the thumb represents the height of the maximum ROM on each shoulder. Note the difference between the adduction and internal rotation of the arms in an athlete with a deficit of 25° of internal rotation. (shown by the arrow).

Internal impingement was assessed with the method described by Meister et al. [8] Tears of the posterior labrum can be detected using this method. The patient is positioned in a supine position with the shoulder placed in 90° of abduction and maximum external rotation (Fig. 3.). The aim of this procedure is to recreate the deep posterior pain at the shoulder.

The SICK syndrome (Scapular malposition, Inferior medial border prominence, coracoid pain and malposition, and dyskinesis of scapular movement) are often seen in patients with GIRD were also assessed by SICK scapula rating scale and physical examination as described by Burkhart et al. Scapular dyskinesia were checked and measured with the method described by Burkhart et al. [9].

Finally, we evaluated rotator cuff strength, shoulder movements (forward flexion, extension, abduction, cross body abduction, Hawkins-neer test), biceps pathology (active compression, Speed test), instability (apprehension test, jerk test).

Consent from the patients for publication and writing the report had been acquired before the examinations. We reported our findings in line with the PROCESS 2018 criteria [10]. Ethical approval was consulted with our internal review board but deemed unnecessary. Our study is registered in a publicly accessible database [11].

## 3 Results

Among the ten athletes (seven males and three females), all were right handed. The athletes had an average age of 18 with average duration of training professionally was 3.8 years. One athlete complained about the pain on the anterior part of her dominant shoulder for 2 months. (see Table 1, Table 2).

Examination of the shoulder revealed that two out of ten athletes (hammer thrower and shot putter) had a deficit of internal rotation on their dominant arm compared with the non-dominant arm. The differences were 20° and 25° respectively. Both subjects had negative Tyler test results.

Three athletes had a positive Tyler's test (posterior shoulder tightness), but no deficit of internal rotation from total range of motion examination on both extremities.

The athlete with complaint of shoulder pain (a female javelin athlete) were examined and revealed a positive biceps pathology, with positive Speed and active compression test on her dominant arm examination.

No athletes had signs of shoulder instability or apprehension-sign, and sulcus sign were negative in all subjects. No subjects had scapular dyskinesia nor SICK scapula syndrome. All athletes had normal shoulder ROM. Using the SICK scapula rating scale by Burkhart, four athletes had score 1–2, no athletes had scapular malposition during the examination.

We observed that all athletes had a mean 6° more external rotation (ER) on their dominant arm when compared with the non-dominant arm (ER mean of 104.5° and 98° respectively).

## 4 Discussion

The throwing motion involves a series of movement that put tremendous stresses on the shoulder joint. Such stresses may elicit shoulder pain and dysfunction in overhead throwing athletes. These complaints had been studied extensively in baseball pitchers but lacking in other branches of sports [2,8,12].

The internal rotation deficit on the shoulder is generally thought as a result of “microtrauma” at the anterior capsule in the cocking phase of throwing. The continuous stretch on the anterior structure of the shoulder will lead to hyperexternal rotation and hyperhorizontal abduction. Burkhart et al. proposed that an acquired internal rotation loss is caused by a posteroinferior capsular contracture secondary to the excessive external rotation [1,2,9].

Cadaveric studies performed by Dillman et al. and Fleisig et al. had shown that instability of the shoulder can lead to degenerative changes in the athlete's shoulder. Repetitive micromotion on the athlete will put permanent changes on the structures around the shoulder [1]. The shoulder is heavily reliant on the labro-capsular-ligamentous complex, along with the rotator cuff muscles [5]. Eventually, these stresses put on the shoulders of the athletes may lead to injury of the shoulders.

Overhead athletes in athletics also share similar fundamentals in the throwing chain thus are comparable with baseball pitchers. The javelin throwing motion only slightly differs from a baseball pitcher's throw, in which the final phase of throw would be a throw beyond the shoulder and a forearm follow through. The pitcher's throw however, has a late cocking, acceleration, and follow through [5,8]. Javelin throwers have been reported to have limited internal rotation just as baseball pitchers. In his report, Schmitt et al. argued that elite javelin athletes injure the rotator cuff, especially the supraspinatus. This injury may lead to anterior-superior translation of the humerus and may attribute to cause the deficit of internal rotation on the shoulder [5].

Setayesh et al. and Copeland explained in their papers the difference regarding the sequence of events in the standard throwing motion from the throws required for hammer throw, discus throw, and shot put [13,14]. The hammer throw involve several spins towards the front of a throwing circle before transferring energy from the lower extremities to the discus or hammer. The discus throw had a “spinning pull” on the disc before the throw [14]. The throw for shot put, entails either two backward steps followed by a spin and release, or a series of spins followed by extension of the elbow, shoulder, and release of the shot put [13].

From our series we found two male athletes (hammer thrower and shot putter) had deficit of internal rotation (IR) of 20 and 25°. However, the two athletes had no posterior shoulder tightness (negative Tyler test). We found three athletes had tightness in their dominant posterior shoulder but no deficit in their internal rotation as shown with the positive Tyler test result. These finding were also found in studies conducted by Tokish et al. and Myers et al. who found no relationship in posterior tightness and GIRD in asymptomatic patients [15,16].

We hypothesized that due to the similar mechanism of the throwing motion of these overhead athletes, similar injury pattern can be obtained but underrecognized. In javelin athletes, the forces and stresses from the throwing and the training program may lead to a reduced internal shoulder rotation with excessive external rotation [17]. The increase of external rotation may be caused by repetitive training external rotation in the cocking phase that increase an eccentric load on the rotator cuff muscles. This excessive external rotation is advantageous to the javelin throwers in that it may increase the acceleration path of the javelin before its release [5,17,18].

Our results however found IR deficit in the hammer thrower and shot putter. In the shot putter, the athlete aimed to release the shot put at a maximum forward velocity with at an angle of approximately forty degrees. The high rotational body speed is gained from the shoulder girdle turning and extension of the right hand [19,20]. In hammer throwers the release of centrifugally induced velocity may lead to chronic rotator cuff tendinopathy and thus contribute to our finding of IR deficit [13,14].

Our series found three athletes had more ER when compared to their non-dominant arm. This may be attributed to adaptive process of at the anterior capsule [2]. A metanalysis by Keller in 2017 showed that total external rotation (ER) may increase the risk of injury when compared with contralateral arm [3]. From his report, he argued that ER gain may place an individual at risk for upper extremity injury.

There are several limitations to this report. First, is the small sample size due to the difficulty of accumulating this unique population. Second, the relative early stage of training period of these athletes which may not reflect the true findings in the population. These athletes had been training in the regional level, but had just recently moved up to national stage. Third, the difference of ROM between joints may be small and within the error of measurement. Finally, it should be noted that this was a group of asymptomatic athletes thus interpretation of the results found must be done in caution.

Further studies using larger sample size and longer observational period to confirm our findings.

## 5 Conclusion

Our series showed two athletes (hammer thrower and shot putter) had internal rotation deficit (20° and 25° respectively) with only five and two years in their respective training as professionals. Coaches, sports medicine doctors, athletes, and the team should be made aware and recognize this problem to avoid or prevent possible further injuries.

## Sources of funding

The authors received no specific funding for this work.

## Provenance and peer review

Not commissioned, externally peer reviewed.

## Funding

No study sponsors were involved in the making of this paper.

## Ethical approval

Ethical approval was consulted and requested with our internal review board but deemed unnecessary by the ethical board.

## Consent

Written informed consent was obtained from the patient for publication of this report and accompanying images.

## Author contribution

AMTL – Study concept and design, data analysis and interpretation, writing the paper.

EIII – Study concept and design, data analysis and interpretation, writing the paper.

NNPPSI, RPW – Data collection, writing the paper.

## Registration of research studies

1. Name of the registry:

Clinical trials.gov.

2. Unique identifying number or registration ID:

ClinicalTrials.gov ID: NCT04418063.

3. Hyperlink to your specific registration (must be publicly accessible and will be checked): https://clinicaltrials.gov/show/NCT04418063.

## Guarantor

The Guarantor is the one or more people who accept full responsibility for the work and/or the conduct of the study, had access to the data, and controlled the decision to publish.

AMTL, EIII, NNPPSI and RPW are the guarantors of this study.

## Declaration of competing interest

The authors have no conflicts of interest to disclose.
